# Evaluation of a Classifier Based on Calprotectin Concentration and Advanced Glycation End-Product Receptor as a Potential Biomarker for Abdominal Aortic Aneurysm

**DOI:** 10.3390/ijms26167752

**Published:** 2025-08-11

**Authors:** Willy Hauzer, Paula Hauzer, Tomasz Klimek, Jan Gnus, Wojciech Witkiewicz, Natalia Jędruchniewicz

**Affiliations:** 1 Department of Vascular Surgery, Hauzer Clinic LLC LP, 55-010 Żerniki Wrocławskie, Poland; 2 Regional Specialist Hospital in Wrocław, Research and Development Centre, 51-124 Wrocław, Poland

**Keywords:** abdominal aortic aneurysm, calprotectin, Receptor for Advanced Glycation End-Products (RAGE)

## Abstract

Calprotectin is a calcium-binding protein involved in inflammatory processes. In the context of abdominal aortic aneurysm (AAA), elevated levels of calprotectin may indicate immune system activation and chronic inflammation, which are among the mechanisms contributing to the development and progression of AAA. The receptor for advanced glycation end-products (RAGE) is a receptor that binds various ligands, including advanced glycation end-products formed during the glycation of proteins and lipids under oxidative stress conditions. Activation of RAGE is associated with inflammatory processes, oxidative stress, and tissue remodeling, which may contribute to the weakening of the aortic wall and aneurysm formation. The main objective of this study was to evaluate the effectiveness of both biomarkers in distinguishing patients with abdominal aortic aneurysm. A total of 27 patients with diagnosed AAA were included in the study. The control group consisted of 27 patients without AAA. Plasma levels of calprotectin and sRAGE were measured in both groups. Statistical analysis included the Shapiro–Wilk test, Mann–Whitney U test, and the Hosmer-Lemeshow (H-L) test. The likelihood of having AAA was found to be over one hundred times greater in individuals classified into the AAA group based on a decision tree model using calprotectin and sRAGE levels, compared to those classified into the no-AAA group. Calprotectin concentration was identified as a stronger predictor of AAA than sRAGE. The optimal cut-off value for plasma calprotectin was determined as ≥1136 ng/mL, yielding a sensitivity of 81.5% and a specificity of 100.0% for discriminating AAA patients from controls. It may be beneficial in future studies to explore non-invasive approaches, such as measuring calprotectin levels in stool and sRAGE in urine, as a potential screening method for AAA. Monitoring the concentrations of these biomarkers in bodily fluids, as a non-invasive method, could support screening efforts for AAA.

## 1. Introduction

### 1.1. Background and Aims

Calprotectin and the receptor for advanced glycation end-products (RAGE) are potential biomarkers for abdominal aortic aneurysm (AAA) [1].

The pathogenesis of abdominal aortic aneurysm (AAA) is complex and multifactorial. It is closely associated with chronic vascular inflammation, progressive degradation of the extracellular matrix, and remodeling of the aortic wall [2,3].

Calprotectin is a calcium-binding protein abundantly expressed in neutrophils and monocytes that plays a central role in inflammatory responses. In the context of AAA, elevated levels of calprotectin may reflect activation of the innate immune system and chronic vascular inflammation, key drivers of aneurysmal degeneration. It activates pro-inflammatory pathways via interaction with pattern recognition receptors such as toll-like receptor 4 (TLR4) and RAGE, leading to nuclear factor kappa-light-chain-enhancer of activated B cells (NF-κB) activation and subsequent release of cytokines including interleukin-6 (IL-6), which has been directly implicated in AAA pathogenesis. Calprotectin is already used as an inflammatory marker in other conditions, such as inflammatory bowel disease, making it an attractive candidate for further investigation in the context of aneurysms [4,5,6].

RAGE is a multiligand receptor of the immunoglobulin superfamily that binds advanced glycation end-products (AGEs), high mobility group box 1 (HMGB1), and S100 proteins including calprotectin. Its activation perpetuates inflammation, oxidative stress, and tissue remodeling—all of which contribute to the weakening of the aortic wall and aneurysm formation. A soluble form of this receptor (sRAGE), which acts as a decoy by sequestering ligands and preventing receptor-mediated signaling, has been proposed as a protective factor. Lower circulating levels of sRAGE have been associated with larger aneurysm diameters and increased systemic inflammation, including elevated calprotectin concentrations. Studies suggest that increased expression of RAGE and its ligands may be linked to AAA pathogenesis. RAGE levels can be measured in different body fluids, such as blood or urine, depending on the research or clinical context [7,8,9,10,11].

Both biomarkers hold potential for use in the diagnosis and monitoring of AAA progression, but further clinical studies are necessary to confirm their utility and specificity. Early detection and monitoring of AAA are crucial for preventing rupture, which is a life-threatening condition [12,13].

In AAA diagnostics, aortic diameter is often considered the “gold standard” for assessment. AAA is typically defined as an enlargement of the aorta exceeding 50% of the normal vessel diameter. Measurement is usually performed using medical imaging techniques such as ultrasound, computed tomography (CT), or magnetic resonance imaging (MRI). Each method has its advantages and limitations, but ultrasound is often preferred for screening due to its availability, safety, and lack of ionizing radiation.

Interestingly, therapeutic strategies under investigation for AAA, such as metformin, may exert part of their beneficial effects through modulation of the AGE–RAGE axis [14,15]. Thus, the interplay between calprotectin and sRAGE reflects the balance between pro-inflammatory and anti-inflammatory processes in AAA, and their combined assessment could enhance diagnostic and prognostic accuracy.

### 1.2. Objective

The primary objective of the study was to evaluate the effectiveness of both biomarkers in distinguishing patients with abdominal aortic aneurysm (AAA) from healthy individuals (control group), analyzed separately and in combination.

## 2. Results

The results of plasma calprotectin and sRAGE concentrations in both groups are presented in Table 1. The table also includes measurements of aneurysm diameter in the AAA group and abdominal aortic diameter in the control group, as aortic diameter assessment using imaging techniques (most commonly ultrasound and computed tomography) is considered the gold standard for both diagnosing and monitoring AAA. Post hoc analyses were conducted to estimate effect size (ES) and statistical power (1-β). The results confirm adequate sample sizes for both study groups. Due to the empirical distributions of the measurement results significantly deviating from a normal distribution, as verified by the Shapiro–Wilk test, the non-parametric Mann–Whitney U test was used for comparisons. These findings suggest that calprotectin alone provides a strong discriminative capacity between AAA and control groups, with an AUC of 0.955.

**Figure 1:**
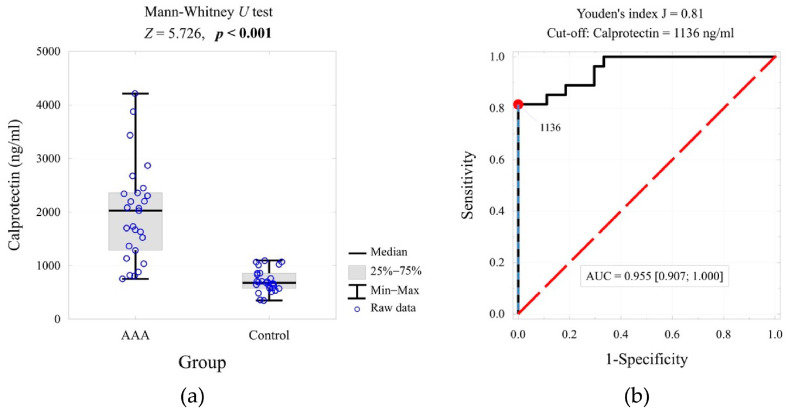
Calprotectin concentration in the compared groups and the result of the significance test (**a**), and the ROC curve for predicting AAA based on the calprotectin concentration, cut-off value, and area under the curve (**b**).

The optimal threshold value for the test based on plasma calprotectin concentration was determined using receiver operating characteristic (ROC) curve analysis (Figure 1). This value (≥1136 ng/mL) was used to construct a contingency table and estimate the odds ratio (Table 2). At this cut-off, calprotectin achieved a sensitivity of 81.5% and specificity of 100.0%, underscoring its strong standalone diagnostic performance.

**Figure 2:**
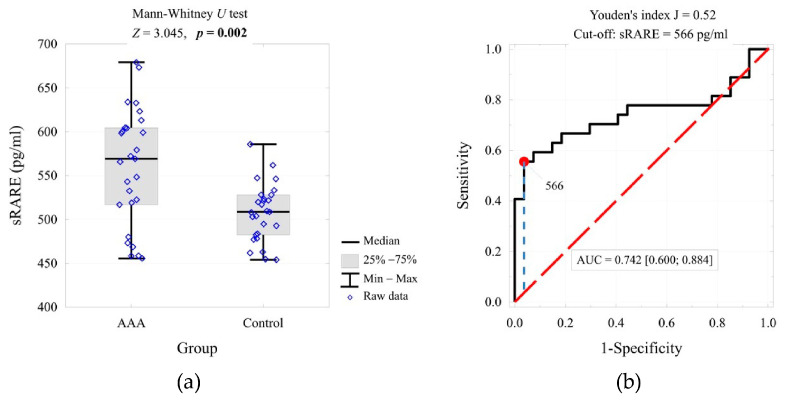
sRAGE concentration in the compared groups and the result of the significance test (**a**), and the ROC curve for predicting AAA based on the sRAGE concentration, cut-off value, and area under the curve (**b**).

The optimal threshold value for the test based on sRAGE concentration was also determined using ROC curve analysis (Figure 2). The identified threshold (≥566 pg/mL) was used for contingency table construction and odds ratio estimation (Table 3). Univariate ROC analysis for sRAGE yielded an AUC of 0.742, with sensitivity of 51.8% and specificity of 96.3% at the chosen cut-off, indicating moderate discriminative power compared to calprotectin.

**Figure 3:**
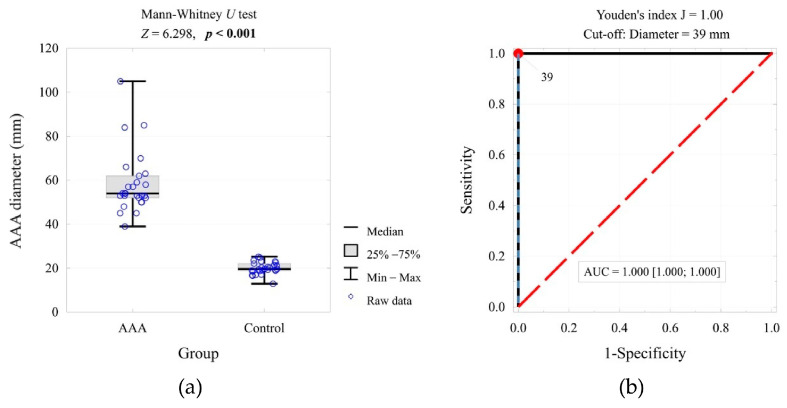
AAA/aortic diameter in the compared groups and the result of the significance test (**a**), and the ROC curve for predicting AAA based on the AAA/aortic diameter, cut-off value, and area under the curve (**b**).

The threshold value for the test based on aortic diameter was established through ROC curve analysis (Figure 3). The value (≥39 mm) was used to build a contingency table and calculate the corresponding odds ratio (Table 4).

**Figure 4:**
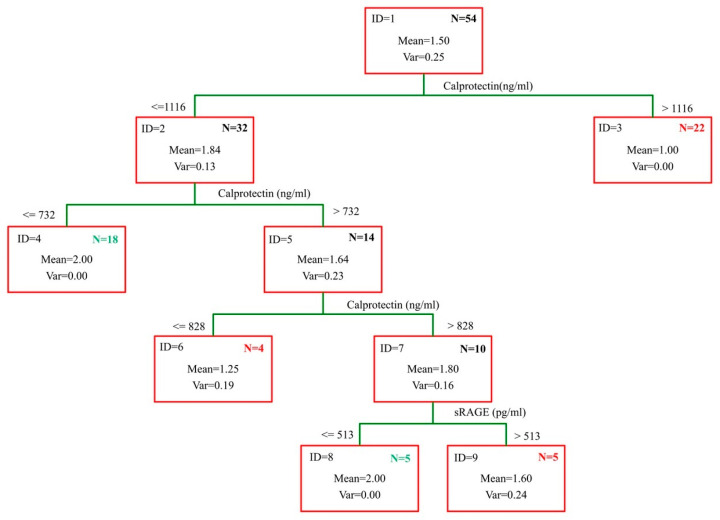
Hierarchical decision tree for AAA identification based on calprotectin and sRAGE levels. Values marked green indicate cases classified as AAA (positive), while those marked red indicate cases classified as no-AAA (negative).

To assess the performance of a classification method based on both biomarkers, a hierarchical decision tree was applied (Figure 4). The tree consists of four decision nodes (ID1, ID2, ID5, and ID7) and five terminal nodes (ID3, ID4, ID6, ID8, and ID9).

**Figure 5:**
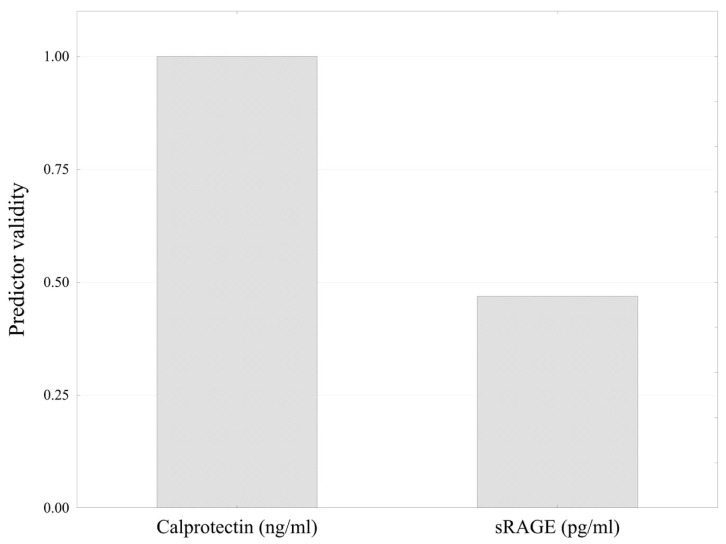
Comparison of biomarker importance in the decision tree.

The importance of each predictor was determined using the Gini importance index during the training of a classification and regression tree model. The importance values reflect the total decrease in node impurity—measured by the Gini index—attributed to each variable across all splits in which the variable was used. Calprotectin contributed approximately twice the reduction in impurity compared to sRAGE, indicating higher predictive importance within the tree-based model (Figure 5).

The contingency table comparing decision tree-based classification against the actual aortic diameter is shown in Table 5.

The decision tree correctly classified 83.3% of participants, indicating its potential utility in clinical screening. The observed OR = 107 confirms the very strong association between biomarker-defined AAA status and true diagnosis.

Decision rule:

IF (Calprotectin > 1116; “AAA”; IF (Calprotectin > 732; “AAA”;
IF (Calprotectin < 828; “no-AAA”; IF (sRAGE > 513; “AAA”; “no-AAA”))))

**Figure 6:**
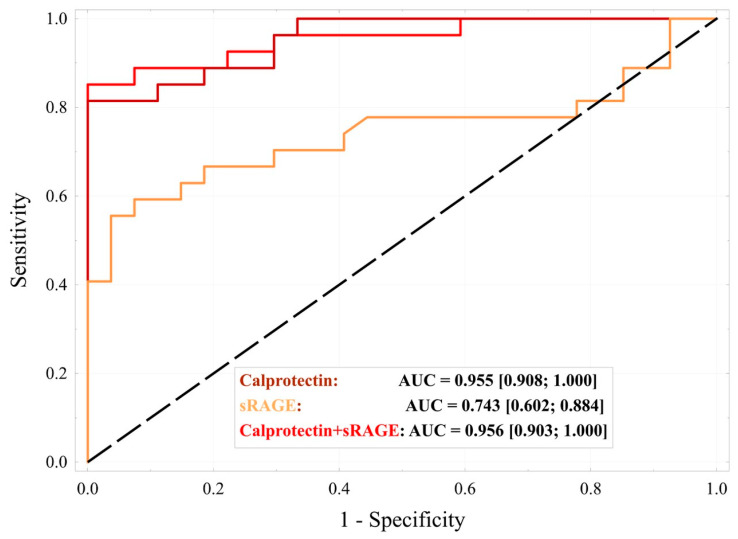
Comparison of ROC curves for AAA prediction using logistic regression models.

An alternative approach to evaluating the performance of both biomarkers involved estimating the probability of AAA using univariate and multivariate logistic regression analyses, in which both biomarkers were treated as continuous variables. The results, along with ROC curves (Figure 6), confirmed that calprotectin concentration is a more effective biomarker than sRAGE. The areas under the curve (AUC) differed significantly (0.955 vs. 0.743, *p* < 0.001). A model combining both markers showed a slightly higher AUC (0.956), but the difference compared to calprotectin alone was not statistically significant (*p* > 0.05).

These results suggest that calprotectin alone provides a strong discriminative capacity between AAA and control groups, with only a marginal AUC improvement when adding sRAGE.

The logistic regression models estimating the probability of AAA based on biomarker concentrations are given below:

- logit (Pr{AAA} = 1|X) = −6.76 + 0.006 × (Calprotectin). H-L = 2.69; *p* = 0.952;
- logit (Pr{AAA} = 1|X) = −10.46 + 0.020 × (sRAGE). H-L = 12.8; *p* = 0.118;
- logit (Pr{AAA} = 1|X) = −19.32 + 0.007 × (Calprotectin) + 0.024 × (sRAGE). H-L = 4.81; *p* = 0.778.

**Figure 7:**
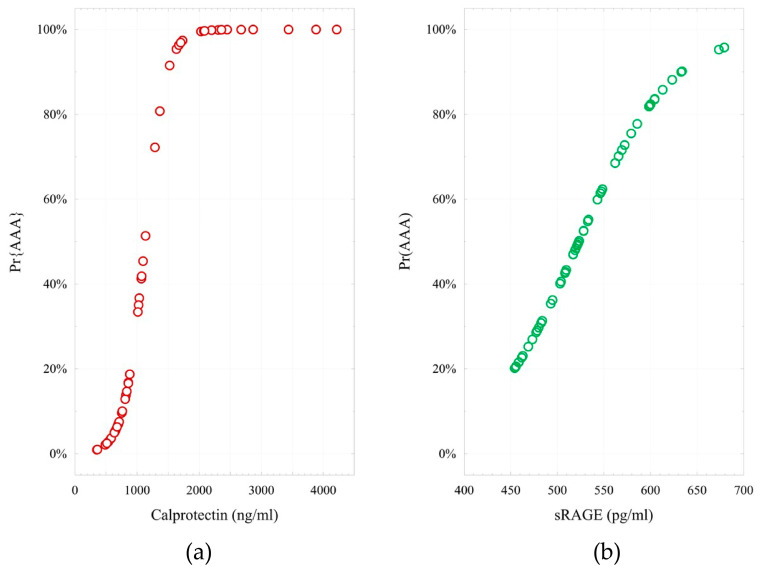
Logistic regression models for predicting AAA based on calprotectin (**a**) and sRAGE (**b**) concentrations.

Model fit to empirical data was evaluated using the Hosmer-Lemeshow (H-L) test. For all three models, the *p*-value was greater than 0.05, indicating good fit, i.e., the models adequately represented the data and the predicted values closely matched the observed outcomes. Figure 7 presents the logistic regression models for predicting AAA based on calprotectin and sRAGE concentrations.

The performance of the decision rules established in the training group of 54 individuals was validated on an independent cohort consisting of 30 patients with AAA and 40 healthy volunteers. The results are presented in Table 6.

Classification performance in the validation group was slightly lower: the odds ratio decreased from OR = 107 in the training group to OR = 61 in the validation group. Specificity decreased from 66.7% to 50.0%, while sensitivity remained at 100%.

The drop in specificity to 50% in the validation cohort highlights possible overfitting of the decision rules and underscores the importance of testing in larger, more diverse populations.

## 3. Discussion

Advanced glycation end-products (AGEs) and their receptor (RAGE) play a crucial role in the pathogenesis of cardiovascular diseases. Among the few available publications on this topic, the study by Fukami et al. [16] deserves particular attention. The authors demonstrated that administration of the soluble form of RAGE (sRAGE) may act as a decoy receptor for AGEs, inhibiting the binding of AGEs to membrane-bound RAGE (mRAGE) and thereby preventing the development and progression of atherosclerosis. Furthermore, the AGEs/High Mobility Group Box 1 (HMGB1)-RAGE axis is involved in heart failure, abdominal aortic aneurysm (AAA), and vascular calcification.

In another study, Chao-Han Lai et al. [17] showed that short-term treatment with recombinant thrombomodulin containing all extracellular domains (rTMD123) suppresses HMGB1–RAGE signaling and provides protection against AAA formation. In an angiotensin II infusion model, a single intravenous injection of recombinant adeno-associated virus vector carrying thrombomodulin lectin-like domain (rAAV-TMD1) (1011 genome copies), which resulted in persistently elevated serum levels of TMD1 for at least 12 weeks, effectively inhibited AAA formation. This was accompanied by suppression of HMGB1 and RAGE levels, reduced production of pro-inflammatory cytokines, macrophage accumulation, matrix metalloproteinase activity, and oxidative stress in the aortic wall.

In another publication, Yao et al. [18] confirmed that RAGE is involved in the development and progression of human AAA. Patients with AAA carrying the RAGE 82S allele variant showed reduced serum levels of soluble RAGE (sRAGE), with the decrease being particularly pronounced in male patients and smokers with AAA. RAGE appears to be a risk factor for AAA. This novel insight may have clinical relevance in the prevention and treatment of AAA.

Zhang et al. [19] further supported the link between RAGE and AAA, indicating that RAGE is associated with age-related diseases, including atherosclerosis, and may also contribute to AAA development.

The role of calprotectin has been explored by Moris et al. [5], who proposed that calprotectin is a novel inflammatory biomarker. Established markers such as C-reactive protein (CRP) and matrix metalloproteinase-9 (MMP-9) were also identified as potential indicators of inflammation in the pathogenesis and progression of AAA. They evaluated the correlation of sRAGE levels with aortic diameter and serum calprotectin in a rat model. The results showed that calprotectin levels in the AAA group significantly increased over time (T14 vs. T7 and T0, *p* < 0.001), whereas sRAGE levels were significantly lower in the AAA group compared to controls across all time points (*p* < 0.001). Calprotectin levels in the AAA group were significantly higher than in controls at T7 and T14 (*p* < 0.001). Aortic diameter was significantly correlated with both serum MMP-9 (*r* = 0.51, *p* < 0.001) and calprotectin (*r* = 0.728, *p* < 0.001), and negatively correlated with sRAGE levels (*r* = −0.48, *p* < 0.001). Additionally, calprotectin and sRAGE were negatively correlated (r = −0.22, *p* < 0.001). The authors highlight that this is the first study to evaluate calprotectin as a potential inflammatory biomarker for AAA. Calprotectin appears to be a promising marker associated with the natural history of AAA. However, further experimental research and large-scale human studies are needed to fully elucidate its role in AAA development and progression.

In another study focused on calprotectin, Plana et al. [6] reported that plasma calprotectin and interleukin-6 (IL-6) levels were significantly elevated in patients with AAA compared to controls (*p* ≤ 0.0001), and a strong correlation between these molecules was observed (*p* < 0.001). Circulating calprotectin may be a specific biomarker for AAA and a potential therapeutic target. It is associated with inflammation and neutrophil activation in the arterial wall and is independent of other atherosclerotic events.

Our findings indicate that plasma calprotectin is a highly promising biomarker for the diagnosis of abdominal aortic aneurysm, demonstrating excellent specificity and strong discriminative power. As a well-established marker of neutrophil-driven inflammation, calprotectin may reflect the underlying chronic inflammatory processes that contribute to aneurysm development and progression. Although sRAGE showed a weaker predictive value in this cohort, its inclusion in the classifier model supported overall diagnostic accuracy, suggesting a potential synergistic role. Importantly, calprotectin’s non-specificity may be offset by its exceptional performance when appropriate exclusion criteria are applied. Future studies should aim to validate these results in larger, more diverse populations and explore the use of fecal calprotectin as a non-invasive tool for early AAA detection and screening.

### Limitations of the Study

While our study design incorporated strict exclusion criteria—specifically omitting participants with inflammatory bowel disease, other acute inflammatory conditions, or exacerbations of chronic diseases—it is important to recognize that calprotectin remains a non-specific marker of inflammation. Plasma concentrations of calprotectin may still be elevated in a variety of subclinical or undiagnosed states, such as mild viral enteritis, low-grade chronic inflammation, or early autoimmune activity, potentially reducing its specificity in real-world populations with higher prevalence of these conditions. Although participants denied the presence of other chronic inflammatory or infectious diseases during structured interviews, no confirmatory diagnostic testing (e.g., CRP, imaging, or microbiological assays) was conducted. This may limit the certainty with which the observed biomarker levels can be attributed exclusively to AAA.

Additionally, although smoking status was verified via self-report and all participants denied current or habitual smoking, no objective biomarker verification was performed. Furthermore, anthropometric measures such as BMI or waist circumference were not collected, which may limit our ability to assess metabolic influences on biomarker levels.

Moreover, our relatively small sample size and the absence of systematic adjustment for key confounders (e.g., smoking status, body mass index) may limit the generalizability of our findings. We also did not compare calprotectin performance directly against other established inflammatory biomarkers, such as C-reactive protein or matrix metalloproteinase-9, which might further delineate its diagnostic utility. Therefore, while calprotectin demonstrated high sensitivity and promising accuracy in our cohort, its clinical applicability as a standalone screening tool for AAA may be constrained. Future research should involve larger, more heterogeneous populations, incorporate comprehensive confounder control, compare calprotectin with additional markers, and consider multi-marker or risk-stratified approaches to enhance both specificity and overall diagnostic performance.

## 4. Materials and Methods

A total of 54 participants were enrolled in the study—27 patients with AAA and 27 control individuals without AAA. All participants were of Caucasian origin and gave written informed consent prior to enrollment. Due to the fact that sex is a known risk factor for AAA, the proportion of men and women was matched between the study and control groups. The general characteristics of the patients in both groups are presented in Table 7.

### 4.1. Study Group

A total of 27 patients with a confirmed diagnosis of AAA were included in the study. All were hospitalized in the Department of Vascular Surgery at the Regional Specialist Hospital in Wrocław, Research and Development Centre (Poland), and admitted for elective AAA surgery. Aneurysm was initially detected by abdominal ultrasound and/or computed tomography and was confirmed intraoperatively. Inclusion criteria: aortic diameter > 30 mm, age > 18 years, and signed informed consent. Exclusion criteria: coexisting malignant disease and dialysis dependency, inflammatory bowel disease, other acute inflammatory conditions or exacerbation of chronic diseases. Upon admission, blood samples were collected to determine plasma levels of sRAGE and calprotectin. A follow-up visit was scheduled three months after the procedure.

### 4.2. Control Group

The control group included 27 individuals without AAA, recruited during “White Saturdays” organized at the Regional Specialist Hospital in Wrocław, Research and Development Centre (Poland). Each participant underwent ultrasound examination confirming the normal course and diameter of the abdominal aorta. Inclusion criteria: Caucasian ethnicity, age > 18 years, normal ultrasound image of the lower abdominal aorta, and signed informed consent. Exclusion criteria: abdominal aortic aneurysm, inflammatory bowel disease, other acute inflammatory conditions or exacerbation of chronic diseases. Participants were selected to match the AAA group in terms of age and the prevalence of comorbidities. No significant differences were observed between groups in age, or in the prevalence of type 2 diabetes, hypertension, or arterial disease (symptomatic peripheral arterial disease of the lower limbs with manifestations of chronic ischemia).

### 4.3. Sample Size

The required sample size for each group (N_1_ = N_2_ = 27) was calculated using G*Power v3.1.9.7 software, based on a significance level of α = 0.05, statistical power of 1-β = 0.80, and expected effect size (ES) of 0.70, as estimated from prior pilot studies.

### 4.4. Study Material

Peripheral blood was collected from the antecubital vein in both the study and control groups using tubes containing an appropriate anticoagulant. In the study group, blood samples were collected prior to surgery. For the determination of calprotectin and sRAGE levels, blood was drawn into EDTA tubes (S-Monovette, 2.9 mL, Sarstedt, Nümbrecht, Germany). Each sample was labeled with a unique code and centrifuged at 1400× *g*. The resulting plasma was aliquoted and stored at −80 °C until biochemical analysis. Plasma concentrations of calprotectin and sRAGE were measured in all participants from both the study and control groups. All analyses were conducted at the Scientific Laboratory of the Regional Specialist Hospital in Wrocław, Research and Development Centre (Poland).

The diameter of the abdominal aorta was measured using B-mode ultrasound imaging during the initial screening. All measurements were taken in the transverse plane, from outer wall to outer wall, by experienced vascular sonographers using a standardized protocol.

### 4.5. Study Methods

The study was conducted in accordance with the Declaration of Helsinki and approved by the Ethics Committee of the Regional Specialist Hospital in Wrocław, Research and Development Centre, Poland (protocol code: KB/6/2017; date of approval: 20 June 2017). All participants included in the study were interviewed and screened for eligibility prior to enrollment. Plasma calprotectin levels were measured using an enzyme-linked immunosorbent assay (ELISA) with a commercially available kit from Assaypro (Saint Charles, MO, USA): AssayMax™ Human Calprotectin ELISA Kit. The assay employed a sandwich format using polyclonal antibodies against human calprotectin and a peroxidase-conjugated detection system. Plasma sRAGE levels were determined using a commercial ELISA kit from Cusabio (Houston, TX, USA): Human Receptor for Advanced Glycation End Products (RAGE/AGER) ELISA Kit. This assay also used a sandwich method with specific antibodies against RAGE and horseradish peroxidase (HRP) as the detection enzyme. All procedures were carried out according to the manufacturers’ instructions. Measurements were performed using the SPECTROstar Nano microplate reader (BMG LABTECH, Ortenberg, Germany), and data were analyzed with MARS Data Analysis Software, version v3.01 R2 (BMG LABTECH, Ortenberg, Germany).

### 4.6. Statistical Analysis

Statistical analysis was conducted using STATISTICA v13.3 and R v4.4.3 (IDE R Studio 25.05.0 Build 496) software. The normality of variable distributions was verified using the Shapiro–Wilk test. As most data did not meet the assumptions of normal distribution, the non-parametric Mann–Whitney U test was applied for group comparisons. Receiver operating characteristic (ROC) curve analysis was used to determine optimal cut-off values. The sensitivity, specificity, and area under the curve (AUC) were calculated. Logistic regression models were used to estimate the probability of AAA based on biomarker levels. Model fit was assessed using the Hosmer–Lemeshow test. Classification accuracy was further evaluated using a decision tree approach. Power analysis and effect size estimates were calculated post hoc.

## 5. Conclusions

Both the decision tree method and logistic regression models confirm that plasma calprotectin concentration is a stronger predictor of abdominal aortic aneurysm (AAA) than sRAGE. The optimal cut-off point for calprotectin concentration (≥1136 ng/mL) yielded a sensitivity of 81.5% and specificity of 100.0% in the training group, and 100% sensitivity with 50% specificity in the validation cohort.

Combining both biomarkers in a single test yields a slight improvement in classification performance; however, this difference is not statistically significant compared to a classifier based solely on calprotectin (*p* > 0.05).

The decision rules derived from the tree analysis could be used to develop a simple AAA risk calculator, for example, in an Excel spreadsheet.

It would also be advisable to measure calprotectin in stool and sRAGE in urine. Monitoring these biomarkers in body fluids, as a non-invasive method, could be useful for AAA screening.

## Figures and Tables

**Table 1:** Basic biomarker statistics in the AAA and control groups and significance test results.

Variable	Group		*p*-Value	ES	1-β
	AAA	Control			
Calprotectin (ng/mL)			<0.001	2.53	1.000
Mean ± SD	1981 ± 902	715 ± 206			
Me [Q1; Q3]	2028 [1286; 2358]	678 [576; 858]			
sRAGE (pg/mL)			0.002	2.16	0.986
Mean ± SD	560 ± 66	508 ± 32			
Me [Q1; Q3]	569 [516; 605]	508 [483; 528]			
AAA/aortic diameter (mm)			<0.001	4.81	1.000
Mean ± SD	58.5 ± 13.9	20.0 ± 2.7			
Me [Q1; Q3]	54 [52; 62]	20 [19; 22]			

Me—median; Q1—lower quartile; Q3—upper quartile; ES—effect size; 1-β—power of the test.

**Table 2:** Number (percentage) of people in groups differing in terms of the presence of AAA and calprotectin concentration, the result of the independence test (Fisher’s exact test), and the odds ratio and its 95% confidence interval.

Calprotectin	Diameter (mm)		Test Result	OR [95% CI]
	≥39 mm *n* = 27	<39 mm *n* = 27		
≥1136 ng/mL	22 (81.5%)	0 (0.0%)	*p* < 0.001 1-β = 1.00	225 [11.8; 4291]
<1136 ng/mL	5 (18.5%)	27 (100.0%)		1.00 (ref.)

ref.—reference category.

**Table 3:** Number (percentage) of people in groups differing in terms of the presence of AAA and sRAGE concentration, the result of the independence test (Fisher’s exact test), and the odds ratio and its 95% confidence interval.

sRAGE	Diameter (mm)		Test Result	OR [95% CI]
	≥39 mm *n* = 27	<39 mm *n* = 27		
≥566 pg/mL	14 (51.8%)	1 (3.7%)	*p* < 0.001 1-β = 1.00	28 [3.31; 236.8]
<566 pg/mL	13 (48.2%)	26 (96.3%)		1.00 (ref.)

ref.—reference category.

**Table 4:** Number (percentage) of people classified by AAA status and aortic diameter, the result of the independence test (Fisher’s exact test), and the odds ratio and its 95% confidence interval.

AAA Status	Diameter (mm)		Test Result	OR [95% CI]
	≥39 mm *n* = 27	<39 mm *n* = 27		
AAA	22 (81.5%)	5 (18.5%)	*p* < 0.001 1-β = 1.00	287 [14.7; 5617]
no-AAA	0 (0.0%)	27 (100.0%)		1.00 (ref.)

ref.—reference category.

**Table 5:** Number (percentage) of patients classified by AAA status (decision tree classification) and aortic diameter, the result of the independence test (Fisher’s exact test), and the odds ratio and its 95% confidence interval.

Decision Tree Classification	Diameter (mm)		Test Result	OR [95% CI]
	≥39 mm *n* = 27	<39 mm *n* = 27		
AAA	27 (100.0%)	9 (33.3%)	*p* < 0.001 1-β = 1.00	107 [5.86; 1955]
no-AAA	0 (0.0%)	18 (66.7%)		1.00 (ref.)

SENS = 100.0%, SPEC = 66.7%, ACC = 83.3%, LR (+) = 3.0; ref.—reference category.

**Table 6:** Number (percentage) of people from the validation group differing in terms of diagnosed abdominal aortic aneurysm and diagnosis made based on rules abstracted from the decision tree.

Decision Tree Rule Diagnosis	AAA *n* = 30	no-AAA *n* = 40	Test Result	OR [95% CI]
AAA	30 (100.0%)	20 (50.0%)	0.001	61.0 [3.49; 1066]
no-AAA	0 (0.0%)	20 (50.0%)		1.00 (ref.)

SENS = 100.0%, SPEC = 50.0%, ACC = 71.4%, LR (+) = 2.0; ref.—reference category.

**Table 7:** Characteristics of patients in the compared groups and group comparison statistics.

Variable	Group		*p*-Value
	AAA	Control	
Age (years), mean ± SD	69.0 ± 6.5	68.7 ± 6.3	0.866
Type 2 diabetes (yes), n (%)	6 (22.2)	4 (14.8)	0.728
Arterial hypertension (yes), n (%)	23 (85.2)	22 (81.5)	1.000
Arterial disease *, n (%)	6 (22.2)	6 (22.2)	1.000

* Symptomatic peripheral arterial disease of the lower limbs with manifestations of chronic ischemia.

## Data Availability

The authors confirm that all data underlying the findings described in this manuscript is fully available to all interested researchers upon request.

## Abbreviations

The following abbreviations are used in this manuscript:

AAA	Abdominal Aortic Aneurysm;
ACC	Accuracy;
AGE	Advanced Glycation End-Product;
AUC	Area Under the Curve;
ES	Effect Size;
H-L	Hosmer–Lemeshow;
HMGB1	High Mobility Group Box 1;
LR	Likelihood Ratio;
mRAGE	Membrane-bound RAGE;
NF-κB	Nuclear factor kappa-light-chain-enhancer of activated B cells
RAGE	Receptor for Advanced Glycation End-Products;
SENS	Sensitivity;
SPEC	Specificity;
sRAGE	Soluble RAGE.
TLR4	Toll-like receptor 4
